# Ethics of the Profession

**Published:** 1904-04

**Authors:** W. A. Summerlin

**Affiliations:** Mt. Vernon, Ga.


### ETHICS OF THE PROFESSION.

I submit to your consideration this question, which has been discussed at so many meetings. From personal experience, I will say that it is the highest pride of the profession to aid those who are suffering and in pain, to give relief regardless of time or cost. One shows the highest respect for the profession when he demonstrates to the public at large that he is above reproach, by his conscientious relations to man, by giving an honest diagnosis at all times, by asking for justly earned fees, and by not showing too much egotism in practice.

In this effort to give services with a high principle, a few become discouraged and drop below the mark of the respectability of truly dignified of the profession. But he who holds out will surely win. It will take time, but alas, be not discouraged, for when you look back upon your path and see the true spirit of the high standard of your career, you can be assured that you will be appreciated by all respectable people, and by the profession, at large. You will find the road rocky, and often be downcast when you remember that Rome, with all the Roman Emperor's zeal helping, was many years in being completed.

From a commercial standpoint, I admit that it is discouraging. We are not speculators, we are not merchants, we are not running department stores. But as we are professional gentlemen, we should follow the dignified principles of the profession, and not even condescend to compete in prices as the department stores do, and we should not deceive the public, as you may rest assured that your sins will find you out sooner or later.

This is a fast age and I fear that if we do not lay down the highest standard, our young men will drift away, and in time will only be as the common tradesmen of the country. We will find so many ignorant of the true spirit of the profession. The public will call to see many and each one gives a different diagnosis, so the public decide for themselves what they wish, and ask that we crown this tooth with a shiner or that one with something else. I say that we should not let the public decide what should be done. When we drift to that point where we are dictated to, it is time to put on brakes.

We often see the clamor for gold so great that sound teeth are sacrificed for the mighty dollar, when a small filling would do just as well. We find a few men that call themselves ethical when they have no idea of the true meaning of the word. They say one thing and practically mean another. They only clamor for gold, and ruin the expression of life for a small fee.

In one town in Georgia, I learned that many young ladies had gold shiners put on their teeth, not that they needed them, but that it is one of the fashions of that town. We see in some sections some of the lady clerks have shiners put on their teeth to attract attention. And still men will put these on for the dollars, regardless of the principle.

I will call your attention to the advertisements of one of the graduates of our college, which reads as follows: "——, D. D. S., will take all kinds of country supplies for services and guarantee all work. Will pay the highest prices in exchange for services rendered."

There are some practitioners who will not consider for a moment the ethical side, or future use of their patients.

I hope the time will come when we can show the public that as a profession, we will not be dictated to by a few fakirs, and a few men who are only imposters on the public, who will do anything for the dollar.

You may rest assured that when the public sees that we have a high motive, it will be duly appreciated.

It is a sad picture to see a man stand for the right, regardless of all cost, when it seems that the fates are against him and the public shows no appreciation for his faithful and honest services, but I ask you, young man, to be faithful and not fall by the roadside. Be men and you will come out all right in the end.—*Georgia State Dental Society Paper, Dr. W. A. Summerlin, Mt. Vernon, Ga.*

